# Antibacterial Activity of Volatile Organic Compounds Produced by the Octocoral-Associated Bacteria *Bacillus* sp. BO53 and *Pseudoalteromonas* sp. GA327

**DOI:** 10.3390/antibiotics9120923

**Published:** 2020-12-18

**Authors:** Anette Garrido, Librada A. Atencio, Rita Bethancourt, Ariadna Bethancourt, Héctor Guzmán, Marcelino Gutiérrez, Armando A. Durant-Archibold

**Affiliations:** 1 Center for Biodiversity and Drug Discovery, Instituto de Investigaciones Científicas y Servicios de Alta Tecnología (INDICASAT AIP), Panama City 0843-01103, Panama; anecgarrido@gmail.com (A.G.); latencio@indicasat.org.pa (L.A.A.); 2 Department of Microbiology and Parasitology, College of Natural, Exact Sciences, and Technology, Universidad de Panama, Panama City 0824-03366, Panama; rita.bethancourt@up.ac.pa (R.B.); ariadna.bethancourt@up.ac.pa (A.B.); 3 Smithsonian Tropical Research Institute, Panama City 0843-03092, Panama; GuzmanH@si.edu; 4 Department of Biochemistry, College of Natural, Exact Sciences, and Technology, University of Panama, Panama City 0824-03366, Panama

**Keywords:** octocoral-associated bacteria, antibacterial activity, volatilome, *Bacillus*, *Pseudoalteromonas*, bacteria volatile organic compounds, antibiotic-resistant bacteria

## Abstract

The present research aimed to evaluate the antibacterial activity of volatile organic compounds (VOCs) produced by octocoral-associated bacteria *Bacillus* sp. BO53 and *Pseudoalteromonas* sp. GA327. The volatilome bioactivity of both bacteria species was evaluated against human pathogenic antibiotic-resistant bacteria, methicillin-resistant *Staphylococcus aureus*, *Acinetobacter baumanni*, and *Pseudomonas aeruginosa*. In this regard, the in vitro tests showed that *Bacillus* sp. BO53 VOCs inhibited the growth of *P. aeruginosa* and reduced the growth of *S. aureus* and *A. baumanni*. Furthermore, *Pseudoalteromonas* sp. GA327 strongly inhibited the growth of *A. baumanni*, and *P. aeruginosa*. VOCs were analyzed by headspace solid-phase microextraction (HS-SPME) joined to gas chromatography-mass spectrometry (GC-MS) methodology. Nineteen VOCs were identified, where 5-acetyl-2-methylpyridine, 2-butanone, and 2-nonanone were the major compounds identified on *Bacillus* sp. BO53 VOCs; while 1-pentanol, 2-butanone, and butyl formate were the primary volatile compounds detected in *Pseudoalteromonas* sp. GA327. We proposed that the observed bioactivity is mainly due to the efficient inhibitory biochemical mechanisms of alcohols and ketones upon antibiotic-resistant bacteria. This is the first report which describes the antibacterial activity of VOCs emitted by octocoral-associated bacteria.

## 1. Introduction

For centuries, the fight against bacterial infections has been one the focus of attention of humanity. Although in the 20th century an important number of discovered antibacterial compounds have improved the quality of life of the people, it has been observed an increased antibiotic resistance prevalence among bacteria which represents the greatest challenge to human health. The main molecular mechanisms of bacteria resistance to antibiotics are due to mutations in bacterial genes; and due to the elevated number of multidrug resistance pumps (MDR pumps), which extrudes antibiotics out of the bacterial cells [1,2,3]. It is therefore imperative the discovery of new therapeutic compounds that overcome the bacterial resistance to antimicrobial agents. Among the major critical group of multidrug-resistant bacteria are *Staphylococcus aureus*, *Pseudomonas aeruginosa*, and *Acinetobacter baumannii* [4]. *S. aureus* is a commensal Gram-positive bacteria, which causes different types of diseases such as endocarditis, septic arthritis, necrotizing fasciitis, parotitis, pyomyositis, osteomyelitis, and skin infections [5]. *P. aeruginosa* is an opportunistic pathogen that is a leading cause of morbidity and mortality in cystic fibrosis patients and immunocompromised individuals. Moreover, it is one of the main risk factors for nosocomial infections and ventilator-associated pneumonia [6]. *A. baumannii* is a Gram-negative *Bacillus*, which is the main cause of hospital-acquired infections, leading to septicemia and pneumonia in immune-compromised hosts [7]. 

Natural antibacterial agents represent the main source of new drugs [8,9]. Most of the scientific investigations for the discovery of drugs with bioactivity against different illnesses have been focused on living organisms from the terrestrial ecosystem. However, in recent years an important number of researches undertaken for the discovery of new bioactive natural products have been performed on marine organisms [10,11]. In this sense, corals (Cnidaria) are aquatic invertebrates in which thousands of bacterial phylotypes coexist. Coral’s microbiome association mainly depends on the corals’ species on which they develop a relevant role in the biosynthesis of compounds with a high degree of bioactivities, for protection against pathogenic microorganisms. Thus, the coral microbiome is an important source of antibacterial natural products [11,12,13,14]. 

Many marine bacteria produce volatile organic compounds (VOCs) whose bioactivity against human pathogenic bacteria are at the early stages of investigation [15,16,17,18,19,20]. These VOCs are small molecules biosynthesized by primary and secondary metabolic pathways and include chemical classes such as alcohols, esters, aliphatic and aromatic hydrocarbons, terpenes, nitrogen, and sulfur compounds, among others. The bacteria volatile organic compounds (bVOCs), contribute to the intra- or inter-communication, and protection against other microorganisms [15,17]. Taking this into account, marine bacteria VOCs can be considered a highly potential source of drugs with antibacterial bioactivity. Despite bacterial symbionts of octocorals represent a source of potential antibiotic drugs [14], no investigation has been focused on the assessment of the antibacterial activity of bVOCs. Accordingly, the purpose of this research is to study the antibiotic activity of VOCs of bacteria isolated from two different octocoral species of the Caribbean Sea against human pathogenic bacteria *P. aeruginosa*, *A. baumannii*, and methicillin-resistant *S. aureus*. 

## 2. Results

### 2.1. Identification of Marine Bacteria BO53 and GA327

The marine bacteria species BO53 and GA327, isolated in Panama from *Pseudopterogorgia acerosa* (Pallas, 1766) and *Muriceopsis sulphurea* (Donovan, 1825), respectively, were identified by 16S rRNA gene sequence analyses. In this sense, the 16S rRNA gene sequence of each species shows 99% sequence similarity with *Bacillus sp.* (BO53) and *Pseudoalteromonas* sp. (GA327) species, when compared to those in the GenBank database (https://www.ncbi.nlm.nih.gov/genbank), using the Basic Local Alignment Search Tool (BLAST). GeneBank accession numbers of the 16S rRNA sequence of BO53 and GA327 are MK291446 and KU213068, respectively.

### 2.2. Antibacterial Activity of VOCs Produced by Bacillus sp. BO53 and Pseudoalteromonas sp. GA327

The in vitro study to determine the antibacterial activity of volatile compounds produced by marine bacteria *Bacillus* sp. BO53 and *Pseudoalteromonas* sp. GA327 species revealed that both bacterial compounds had inhibitory activity towards *A. baumannii*, *P. aeruginosa*, and methicillin-resistant *S. aureus* growth (Table 1). The Gram-positive *Bacillus* sp. BO53 volatile organic compounds lead to significant growth inhibition of *P. aeruginosa* at 24 h, and to growth reduction of *S. aureus* and *A. baumannii* at 48 h. The volatiles released by the Gram-negative *Pseudoalteromonas* sp. GA327 lead to the growth inhibition of *A. baumannii* at 24 h, and *P. aeruginosa* at 48 h, and did not inhibit the growth of *S. aureus*. On the other hand, in the absence of bVOCs produced by marine bacteria, the growth of human pathogenic bacteria was not suppressed. The inhibition of Gram-negative pathogenic bacteria growth by marine bacteria VOCs was greater for *Pseudoalteromonas* sp. GA327, than *Bacillus* sp. BO53; while on the other hand, the bioactivity of VOCs on Gram-positive pathogenic bacteria, was higher for *Bacillus* sp. BO53 than *Pseudoalteromonas* sp. GA327.

### 2.3. Identification of VOCs from Bacillus sp. BO53 and Pseudoalteromonas sp. GA327

The analysis for identification of VOCs biosynthesized by octocoral-associated bacteria *Bacillus* sp. BO53 and *Pseudoalteromonas* sp. GA327 was performed by HS-SPME-GC-MS technique. Each marine bacteria sample was analyzed three times using the DVB/CAR/PDMS coating solid-phase microextraction fiber; 37 °C extraction temperature; and 40 min extraction time. DVB/CAR/PDMS fiber was selected due to its high capacity for the extraction of volatile and semi-volatile compounds present in samples [21,22,23].

**Figure 1:**
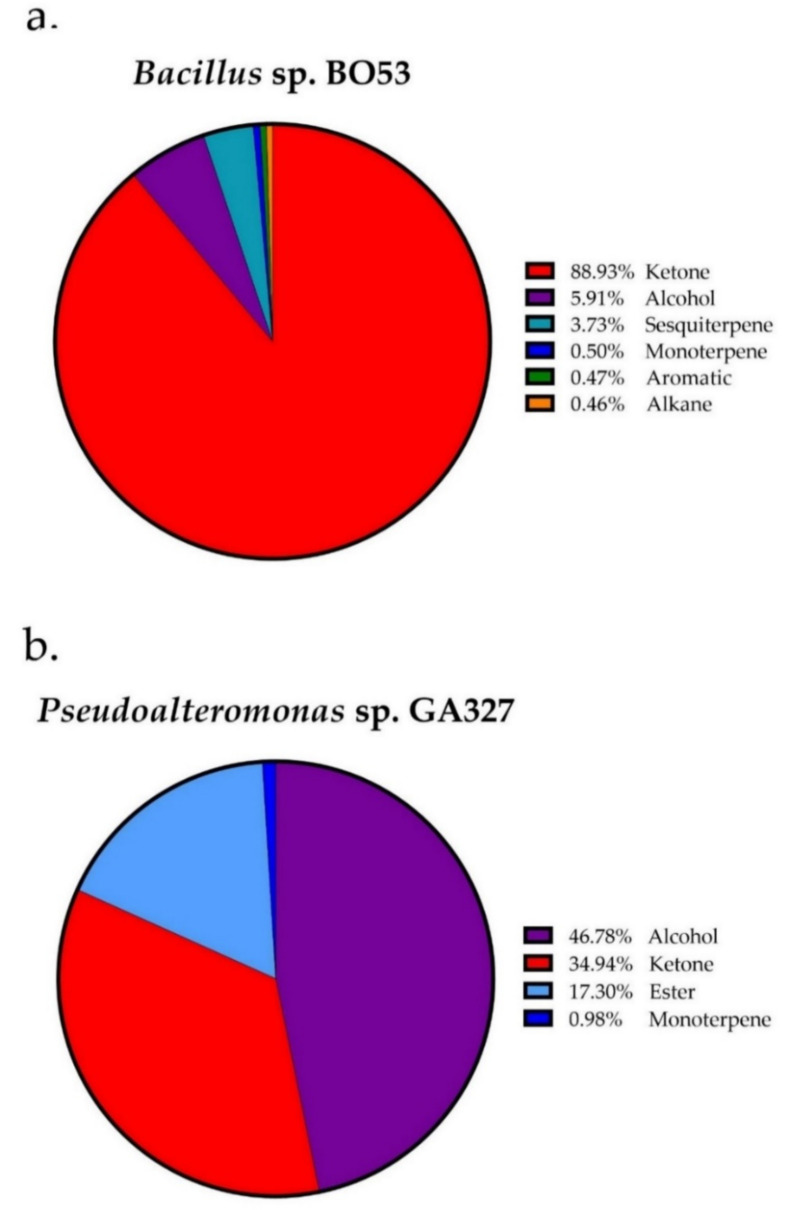
The proportion of the chemical families of VOCs detected in octocoral-associated bacteria *Bacillus* sp. BO53 and *Pseudoalteromonas* sp. GA327.

**Figure 2:**
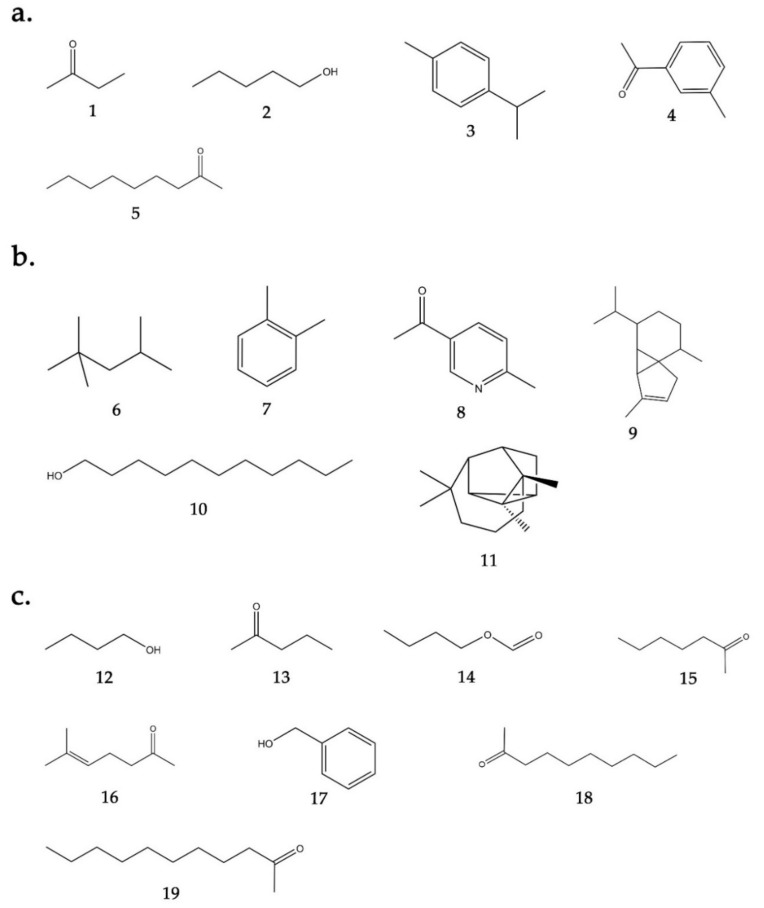
Molecular structure of *Bacillus* sp. BO53 and *Pseudoalteromonas* sp. GA327. detected volatile compounds. (**a**) VOCs detected on *Bacillus* sp. BO53 and *Pseudoalteromonas* sp. GA327: (1) 2-butanone, (2) 1-pentanol, (3) p-cymene, (4) 3-methylacetophenone, (5) 2-nonanone, (**b**) VOCs detected only on *Bacillus* sp. BO53: (6) 2,2,4-trimethylpentane, (7) o-xylene, (8) 5-acetyl-2-methylpyridine, (9) α-cubebene, (10) 1-decanol, (11) logicyclene, (**c**) VOCs detected only on *Pseudoalteromonas* sp. GA327: (12) 2-butanone, (13) 2-pentanone, (14) butyl formate, (15) 2-heptanone, (16) 6-methyl-5-heptene-2-one, (17) benzyl alcohol, (18) 2-decanone, (19) 2-undecanone.

In total, 19 bVOCs were identified, of which 11 were detected in *Bacillus* sp. BO53 and 13 in *Pseudoalteromonas* sp. GA327 (Table 2). Compounds that were present in the blank Luria Bertani (LB) broth, supplemented with seawater, were excluded. Ketone comprised the largest compounds detected in *Bacillus* sp. BO53, followed by alcohols, sesquiterpenes, monoterpenes, aromatics, and alkanes (Figure 1a). 5-Acetyl-2-methylpyridine (64.63%), 2-butanone (17.03%), and 2-nonanone (7.00%) were the major VOCs detected in *Bacillus* sp. BO53. In the case of *Pseudoalteromonas* sp. GA327, alcohols are the main compounds detected followed by ketones, ester, and monoterpene compounds (Figure 1b). 1-Pentanol (38.91%), 2-butanone (20.14%), and butyl formate (17.30%) were the primary VOCs detected in *Pseudoalteromonas* sp. GA327. 2,4-trimethylpentane, o-xylene, 5-acetyl-2-methylpyridine, α-cubebene, 1-undecanol, and α-longicyclene are the specific VOCs produced by *Bacillus* sp. BO53 (Figure 2b); while 1-butanol, 2-pentanone, butyl formate, 2-heptanone, 6-methyl-5-heptene-2-one, benzyl alcohol, 2-decanone, 2-undecanone were found only in *Pseudoalteromonas* sp. GA327 (Figure 2c).

## 3. Discussion

Human pathogenic bacteria, *A. baumannii*, methicillin-resistant *S. aureus,* and *P. aeruginosa* bacteria, are antibiotic-resistant microorganisms for which the development of research for the discovery of new antibacterial drugs have become crucial. To date, only a small number of marine bacteria have been studied for bioactive bVOCs [24]. 

This study aimed to determine the antibacterial activity of the volatilome produced by marine bacteria, *Bacillus* sp. BO53 and *Pseudoalteromonas* sp. GA327, isolated from octocorals. Overall, the VOCs produced by *Pseudoalteromonas* sp. GA327 lead to the inhibition of the two Gram-negative pathogenic bacteria investigated at early stages (24 h) when compared to the antibacterial activity of *Bacillus* sp. BO53. The antibacterial activity of *Bacillus* sp. BO53 VOCs against *P. aeruginosa* was higher than for *A. baumannii* and *S. aureus*, and lead to a total inhibition of *P. aeruginosa* growth within 48 h after exposure to the *Bacillus* sp. BO53 VOCs. 

The HS-SPME-GC-MS analysis, lead to the identification of ketones, mainly 5-acetyl-2-methylpyridine, as the most abundant volatile compound produced by *Bacillus* sp. BO53. Among the main VOCs biosynthesized by bacteria are ketones and alcohols [25]. It is evident from this research that the volatile compound 5-acetyl-2-methylpyridine leads to the growth reduction of *A. baumannii*, *S. aureus*, and the inhibition of *P. aeruginosa.* In this sense, pyridine derivatives have shown relevant bioactivity against Gram-positive and Gram-negative bacteria [26]. The antibacterial activity of this volatile compound has to be performed to corroborate its bioactivity. On the other hand, studies have reported a relevant inhibitory activity of 2-butanone upon *S. aureus*, *P. aeruginosa,* and *E. coli* [27]. Furthermore, Arambula et al. [28] have reported an important growth inhibition of *S. aureus* and *E. coli* by 2-nonanone. The alcohol volatile compound 1-undecanol, which was determined as one of the main compounds produced by *Bacillus* sp. BO53 inhibits *S. aureus* by damaging the bacterial cell membrane [29]. This reported bioactivity also might contribute to the growth reduction of methicillin-resistant *S. aureus* observed in the current study. The lack of complete inhibition of *S. aureus* and *A. baumannii* strains can be attributed to the low amount of VOCs produced by *Bacillus* sp. BO53, which was due to its low growth rates. 

The results of this investigation have revealed an effective antibacterial activity of *Pseudoalteromonas* sp. GA327 VOCs on *A. baumannii* and *P. aeruginosa* strains. These observations suggest that alcohol, ketone, and ester volatile compounds, which were the most abundant VOCs identified from *Pseudoalteromonas* sp. GA327, generate an efficient inhibitory biochemical mechanism on the Gram-negative bacteria studied. 1-Pentanol and benzyl alcohol, which were detected in high amounts in *Pseudoalteromonas* sp. GA327, affect the bacterial cell membrane, causing fluidization or interrupting the functions of the membrane proteins. The alteration of the bacterial membrane due to volatile alcohols allows other antimicrobial compounds to easily penetrate the cell membrane [30] 2-Butanone was the main antibacterial ketone identified from *Pseudoalteromonas* sp. GA327. On the other hand, it has been reported that 2-heptanone, 6-methyl-5-heptene-2-one, and 2-undecanone ketones, produced by *Pseudoalteromonas* sp. GA327, present antibacterial properties against pathogenic Gram-positive and Gram-negative bacteria [31,32,33,34,35]. Regarding the inhibitory activity of the volatile ester butyl formate, one of the VOCs detected in higher amounts in *Pseudoalteromonas* sp. GA327, Calvo et al. [36] reported the presence of this molecule within the antifungal VOCs produced by the bacteria *B. velezensis*. Therefore, the results of the present study suggest the potential antibacterial bioactivity of butyl formate. 

## 4. Materials and Methods

### 4.1. Bacterial Isolation from Octocorals

Isolated bacteria GA327 and BO53 were obtained from two octocorals hosts located in coastal Caribbean Sea waters of Panama: GA327 from *Pseudopterogorgia acerosa* and *Muriceopsis sulphurea*. *M. sulphurea* was collected at Punta Galeta in Colon Province (9°24′16′ N 79°51′35″ W), and *P. acerosa* from San Cristobal Island in Bocas del Toro Province (9°15′31″ N 82°16′12″ W). 

For isolation of the octocoral-associated bacteria, 0.5 mL of the coral mucus was inoculated on agar plates with seawater-based nutrient medium (500 mg of mannitol, 100 mg of peptone, 8 g of Noble agar, and rifampicin [5 µg/mL] in 1 L of seawater). The octocoral-associated bacteria, GA327 and BO53, were subsequently isolated from the collection plate and successively replated until the pure isolated bacteria was obtained.

### 4.2. Molecular Identification of Octocoral-Associated Bacteria Species

The genetic identification of the bacterial species GA237 and BO53 was performed based on the methodology described by Atencio et al. [13]. Briefly, for DNA extraction, one milliliter of the GA237 and BO53 species were cultured on Luria Bertani (LB) broth (Difco, Michigan, MI, USA), supplemented with seawater, and grown at 25 °C for 24 h. The samples were then centrifuged at 10,000 rpm for 2 min. The resulting pellet was resuspended in 500 µL of 5% Chelex-100. Each suspension was vortexed and incubated at 56 °C for 20 min, then boiled at 100 °C for 10 min, and placed on ice for 2 min. The samples were centrifuged at 13,000 rpm for 5 min. Subsequently, the supernatants containing the DNA were transferred to a new tube and stored at −20 °C.

The DNA fragment of the 16S rRNA gene was amplified by PCR using primers pairs 27F (5′-AGAGTTTGATCMTGGCTCAG-3′) and 1492R (5′-TACGGYTACCTTGTTACGACTT-3′), and sequenced using 518F (5′CCAGCAGCCGCGGTAATACG3′) and 800R (5′TACCAGGGTATCTAATCC3′) primers [37]. The obtained sequences were compared to 16S rRNA gene sequences, using the BLAST algorithm, deposited in the GenBank, keeping a maximum of 100 hits per query sequence. Moreover, 16S rRNA sequences were compared against RDP (Ribosomal Database Project) [38] and aligned against the SILVA reference database using SINA with a 98% similarity threshold [39]. The nucleotide sequence of the BO53 and GA327 species have been submitted to the GenBank database under the accession number MK291446 and KU213068, respectively.

### 4.3. Pathogenic Bacterial Strains

Pathogenic bacteria *A. baumannii* (ATCC 19606), *P. aeruginosa* (ATCC 10145), and *S. aureus* (ATCC 43300) were maintained on LB medium at 37 °C. Each pathogenic strain was transferred to LB broth and was grown at 37 °C overnight. These broths were used to prepare dilutions of 0.5 McFarland to use as inoculum for the antibacterial activity assays. 

### 4.4. Antibacterial Activity of Marine bVOCs

The antibacterial activity of the VOCs of BO53 and GA327 species were determined by the double plate test method of Romoli et al. [40] with slight modifications. BO53 and GA327 species were cultured by triplicate on LB broth, supplemented with sterile seawater, and incubated at 37 °C for 24 h. A dilution of the cultured marine bacteria was made to achieve a turbidity of 0.5 McFarland, and then each dilution was inoculated on LB plates supplemented with sterile seawater (hereafter marine bacteria plate) and placed on an incubation chamber at 37 °C overnight. Afterward, the Petri dish lid was taken off and a plate with only LB medium (hereafter pathogenic bacteria plate) was placed over the marine bacteria plate. Both plates were sealed with parafilm and incubated at 37 °C for 24 h, to allow the VOCs generated by the marine bacteria to be absorbed in the pathogenic bacteria plate. Afterward, the 0.5 McFarland dilutions of each pathogenic strain were inoculated homogeneously on the pathogenic bacteria plate with a sterile cotton swab, and placed again over the marine bacteria plate, sealed with parafilm, and incubated at 37 °C for 48 h. The pathogen’s growth was evaluated every 24 h. Antibacterial activities were compared to negative controls. The pathogenic bacteria growth inhibition by the marine bVOCs was judged as “+” (complete inhibition), “±” (reduced growth), and “-“ (no detectable bioactivity). Each experiment was carried out in triplicate.

### 4.5. Marine Bacteria Volatolome Analysis

*Pseudoalteromonas* sp. GA327 and *Bacillus* sp. BO53 species were cultured in glass vials, by triplicate, on LB medium supplemented with sterile seawater and incubated at 37 °C for 24 h. The samples were subsequently analyzed after 48 h of incubation. Three vials containing LB medium supplemented with sterile seawater, but not inoculated, were incubated under the same conditions.

The VOCs of all samples were analyzed by headspace-solid phase microextraction-gas chromatography-mass spectrometry (HS-SPME-GC-MS) method [22,41]. A divinylbenzene-carboxen-polydimethylsiloxane (DVB/CAR/PDMS 50/30 µm) fiber (Supelco, Bellefonte, PA, USA) was exposed to the headspace of the samples for 40 min at 37 °C. The isolated VOCs were analyzed by GC-MS, on a GC 6890N coupled to a 5975C mass spectrometry detector (Agilent Technologies, Palo Alto, CA, USA). VOCs were desorbed by insertion of the SPME fiber into the GC injection port, in splitless mode, for 2 min at 250 °C. The compounds were separated on an HP-5MS capillary column (30 m length, 0.25 mm id, 0.25 µm), using He as carrier gas at 1 mL/min. The oven temperature was 50 °C for 2 min, then increased to 240 °C at 6 °C/min and held for 5 min. MS detector was operated in electron impact mode (EV = 70 eV); in scan mode from 30 to 550 *m*/*z*; with an ion source temperature of 250 °C.

VOCs were identified by comparing their MS spectra with Registry of Mass Spectral Data with Structures library (Wiley 7th edition, USA), and National Institute of Standards and Technology library (NIST) spectral databases, and by using authentic standards when available. Additional identification was performed by determination of the compounds Kovat’s retention index (RI) by using an alkane standard solution C8-C20 (Sigma- Aldrich, Saint Louis, MO, USA). VOCs compounds identified in vials not inoculated were excluded from the data analyses. The relative quantities of the volatile compounds are expressed as percent peak areas relative to the total peak area of identified compounds from the average of the three replicates [22,42].

## 5. Conclusions

The antibacterial activity of octocoral-associated bacteria *Bacillus* sp. BO53 and *Pseudoalteromonas* sp. GA327 VOCs were determined for the first time. *Bacillus* sp. BO53 volatile compounds lead to complete inhibition of *P. aeruginosa* and displayed growth reduction on *A. baumannii* and methicillin-resistant *S. aureus*; while *Pseudoalteromonas* sp. GA327 VOCs exhibited a high inhibition against both Gram-negative bacteria species and were inefficient against *S. aureus* growth. HS-SPME-GC-MS methodology allowed the identification of VOCs produced by both octocoral-associated bacteria. Alcohol and ketone volatile compounds were the most abundant VOCs detected. The bacterial emission of these VOCs might explain the antibacterial activity observed. The results of this study justified future research to determine the antibacterial activity of a few of the identified VOCs to evaluate their potential bioactivity against antibiotic-resistant bacteria.

## Figures and Tables

**Table 1:** Antibacterial effect of *Bacillus* sp. and *Pseudoalteromonas* sp. volatile compounds against *Acinetobacter baumanni*, *Staphylococcus aureus*, and *Pseudomonas aeruginosa* pathogenic bacteria.

Species	Strain	*Bacillus* sp. BO53		*Pseudoalteromonas* sp. GA327	
		24 h	48 h	24 h	48 h
*A. baumanni*	ATCC 19606	-	±	+	+
*S. aureus*	ATCC 43300	-	±	-	-
*P. aeruginosa*	ATCC 10145	-	+	±	+

(+) Growth inhibition; (±) Growth reduction; (-) No inhibition.

**Table 2:** Identified mVOCs produced by the marine bacteria *Bacillus* sp. BO53 and Pseudoalteromonas sp. GA327.

			% Detected Compound on Each Species	
Compound	TRI	ERI	*Bacillus* sp. BO53	*Pseudoalteromonas* sp. GA327
2-Butanone	602	609	17.03	20.14
1-Butanol	671	676	-	4.55
2,2,4-Trimethylpentane	680	687	0.46	-
2-Pentanone	687	693	-	1.29
1-Pentanol	775	780	1.33	38.91
Butyl formate	787	793	-	17.30
o-Xylene	884	891	0.47	-
2-Heptanone	889	895	-	7.99
6-Methyl-5-heptene-2-one	988	995	-	0.37
p-Cymene	1021	1027	0.50	0.98
Benzyl Alcohol	1033	1040	-	3.32
2-Nonanone	1096	1102	7.00	1.38
3-Methylacetophenone	1176	1184	0.26	1.18
5-Acetyl-2-methylpyridine	1189	1193	64.63	-
2-Decanone	1190	1198	-	1.01
2-Undecanone	1291	1300	-	1.58
α-Cubebene	1354	1355	0.43	-
1-Undecanol	1370	1374	4.58	-
α-Longicyclene	1374	1380	3.30	-

TRI: Theoretical Retention Index; ERI: Experimental Retention Index.
